# Principles of Human Physiology, with Their Chief Applications to Pathology, Hygiene and Forensic Medicine

**Published:** 1847-10

**Authors:** 


***Principles of Human Physiology, with their chief applications to Pathology, Hygiene and Forensic Medicine.*** By Wm. B. Carpenter, M. D., F. R. S., &c. &c. &c. Third American, from the last London edition, with notes and additions by Meredith Clymer, M. D. With 317 wood cuts and other illustrations. Philadelphia: Lea & Blanchard.

The character of Dr. Carpenter's Physiology is too well known to require any exhibition by us. It is a thorough work on this interesting and important subject, and having passed through three editions in Great Britain and in this country, it may be considered as established in the public regard. To all who desire to make themselves acquainted with Human Physiology we recommend this work.

---

### Miscellaneous Notices.

